# The CANNA-TICS Study Protocol: A Randomized Multi-Center Double-Blind Placebo Controlled Trial to Demonstrate the Efficacy and Safety of Nabiximols in the Treatment of Adults With Chronic Tic Disorders

**DOI:** 10.3389/fpsyt.2020.575826

**Published:** 2020-11-26

**Authors:** Ewgeni Jakubovski, Anna Pisarenko, Carolin Fremer, Martina Haas, Marcus May, Carsten Schumacher, Christoph Schindler, Sebastian Häckl, Lukas Aguirre Davila, Armin Koch, Alexander Brunnauer, Camelia Lucia Cimpianu, Beat Lutz, Laura Bindila, Kirsten Müller-Vahl

**Affiliations:** ^1^Clinic of Psychiatry, Social Psychiatry and Psychotherapy, Hannover Medical School, Hannover, Germany; ^2^Clinical Research Center Core Facility, Hannover Medical School, Hannover, Germany; ^3^Center for Pharmacology and Toxicology, Hannover Medical School, Hannover, Germany; ^4^Hannover Medical School, Institute for Biostatistics, Hannover, Germany; ^5^Section Biostatistics, Paul-Ehrlich-Institute, Langen, Germany; ^6^Department of Neuropsychology, Kbo-Inn-Salzach-Klinikum, Psychiatric Hospital, Wasserburg am Inn, Germany; ^7^Department of Psychiatry and Psychotherapy, Ludwig-Maximilians University Munich, Munich, Germany; ^8^Institute of Physiological Chemistry, University Medical Center of the Johannes Gutenberg University, Mainz, Germany

**Keywords:** cannabidiol, THC, tetrahydrocannabinol, cannabinoids, nabiximols, chronic tic disorder, tics, tourette syndrome

## Abstract

**Background:** Gilles de la Tourette syndrome (TS) is a chronic neuropsychiatric disorder characterized by motor and vocal tics. First-line treatments for tics are antipsychotics and tic-specific behavioral therapies. However, due to a lack of trained therapists and adverse events of antipsychotic medication many patients seek alternative treatment options including cannabis. Based on the favorable results obtained from case studies on different cannabis-based medicines as well as two small randomized controlled trials using delta-9-tetrahydrocannabinol (THC), we hypothesize that the cannabis extract nabiximols can be regarded as a promising new and safe treatment strategy in TS.

**Objective:** To test in a double blind randomized clinical trial, whether treatment with the cannabis extract nabiximols is superior to placebo in patients with chronic tic disorders.

**Patients and Methods:** This is a multicenter, randomized, double-blind, placebo controlled, parallel-group, phase IIIb trial, which aims to enroll 96 adult patients with chronic tic disorders (TS or chronic motor tic disorder) across 6 centers throughout Germany. Patients will be randomized with a 2:1 ratio into a nabiximols and a placebo arm. The primary efficacy endpoint is defined as tic reduction of at least 30% (compared to baseline) according to the Total Tic Score of the Yale Global Tic Severity Scale (YGTSS-TTS) after 13 weeks of treatment. In addition, several secondary endpoints will be assessed including changes in different psychiatric comorbidities, quality of life, driving ability, and safety assessments.

**Discussion:** This will be the first large, controlled study investigating efficacy and safety of a cannabis-based medicine in patients with TS. Based on available data using different cannabis-based medicines, we expect not only a reduction of tics, but also an improvement of psychiatric comorbidities. If the cannabis extract nabiximols is proven to be safe and effective, it will be a valuable alternative treatment option. The results of this study will be of high health-economic relevance, because a substantial number of patients uses cannabis (illegally) as self-medication.

**Conclusion:** The CANNA-TICS trial will clarify whether nabiximols is efficacious and safe in the treatment of patients with chronic tic disorders.

**Clinical Trial Registration:** This trial is registered at clinicaltrialsregister.eu (Eudra-CT 2016-000564-42) and clinicaltrials.gov (NCT03087201).

## Introduction

Gilles de la Tourette syndrome (TS) is a common, complex, chronic neuropsychiatric disorder characterized by motor and vocal tics. It causes not only significant impairment in quality of life of the affected patients, but also significant economic costs in the German health care system as a whole (1, 2). The treatment options for chronic tic disorders (CTD) and TS are limited: to date haloperidol is the only approved drug in Germany, which is barely prescribed any more due to severe adverse events (AEs) (3). Instead, most clinicians prefer an off-label use of other antipsychotics such as aripiprazole and risperidone. However, due to AEs and/or lack of efficacy, a substantial number of patients is unsatisfied with this kind of treatment. First-line behavioral therapies (BT) such as Habit Reversal Training (HRT) and Exposure with Response Prevention (ERP) are not available to most patients, because of poor dissemination of these therapy techniques among psychotherapists (3, 4). Therefore, many patients with CTD are looking for complementary and alternative medicine (CAM) including self-medicating with cannabis (5).

Until today only a small number of case studies and series (all together including about 200 patients) is available, reporting about beneficial effects of different cannabis-based medicines including pure delta-9-tetrahydrocannabinol (THC, dronabinol), cannabis extracts, and cannabis flowers in patients with TS. Interestingly, in most of these studies not only a tic reduction is reported, but also an improvement of a broad spectrum of psychiatric comorbidities including attention deficit/hyperactivity disorder (ADHD), obsessive compulsive disorder (OCD), depression, anxiety, rage attacks, sleep disorders, and self-injurious behavior resulting in a significant improvement of patients' quality of life. Most interestingly, in some of these cases, in addition, an improvement of premonitory urges proceeding the occurrence of a tic is described [for review see (6)].

So far only two small randomized controlled trials (including 12 and 24 patients, respectively), have been carried out to further investigate the treatment effects of cannabis-based medicines in patients with TS. In both of these studies pure THC, the most psychoactive ingredient of cannabis, has been used. According to these studies, THC resulted in a tic reduction and was well-tolerated without causing severe AEs or relevant neuropsychological impairment (7, 8).

This study aims to further examine the efficacy and safety of cannabis-based medicines in patients with CTD. At the time, when this study was designed, the only cannabis-based medicines that could theoretically be used in clinical trials in Germany were pure THC, the synthetic THC-analog nabilone, cannabis flowers, and the cannabis extract nabiximols. We decided to use nabiximols, a plant extract from Cannabis sativa L. that contains THC and cannabidiol (CBD) at a 1:1 ratio, for the following reasons: (i) at that time, nabiximols was the only officially licensed cannabis-based medicine in Germany (since 2010 licensed for the treatment of spasticity in multiple sclerosis) (9), (ii) compared to pure THC, it can be assumed that nabiximols – according to the so called entourage effect - is not only more effective [since CBD possesses its own effects (10)], but also better tolerated [since co-administration of CBD mitigates unwanted psychotropic effects of THC (11)], and (iii) compared to herbal products, for most patients preparation, application and intake is easier. Finally, GW Pharma Ltd. kindly agreed to offer nabiximols and placebo as investigational medical product (IMP) for this investigator-initiated study.

The introduction of a prescription of cannabis flowers in Germany in 2017 went along with an intensive and controversial debate on whether treatment with cannabis-based medicines may have a negative impact on patients' driving ability and whether patients should be allowed to drive a car. While in the case of recreational use of cannabis, driving a car is generally not allowed as long as THC tests are positive, the German government stated that in contrast cannabis-based medicines - when prescribed and supervised by a medical doctor - should be handled comparably to other psychoactive drugs (12). This implies that the subject is responsible to accurately self-assess his or her driving ability before using a vehicle. With respect to nabiximols, it has been shown that driving ability is not impaired in patients with multiple sclerosis (13, 14). However, in patients with TS so far only one single case study has been published reporting an improvement of patient's driving ability after treatment with THC (15). To increase our current knowledge regarding the effects of cannabis-based medicines on driving ability specifically in patients with TS, we plan to perform tests of driving ability at baseline and after treatment with nabiximols.

This study is designed as a multicenter randomized double-blind placebo controlled trial using nabiximols compared to placebo. We plan to include 96 adult patients at six large specialized TS centers all across Germany. The effects of nabiximols on tics, comorbidities, and patients' driving ability as well as AEs will be assessed during and after treatment.

## Methods and Analysis

### Drug Information

Nabiximols is a complex botanical mixture derived from the *Cannabis sativa* plant. It contains different cannabinoids and terpenes with THC and CBD being the most abundant cannabinoids present. Nabiximols is a sublingually administered oromucosal spray that contains 10 ml solution in one spray container. The containers have to be stored in accordance with the German legal requirements [German Narcotic Drugs Act (“Betäubungsmittelgesetz,” BtMG)]. Nabiximols and placebo will be manufactured by GW Pharma Ltd., United Kingdom.

### Study Design

**Figure 1:**
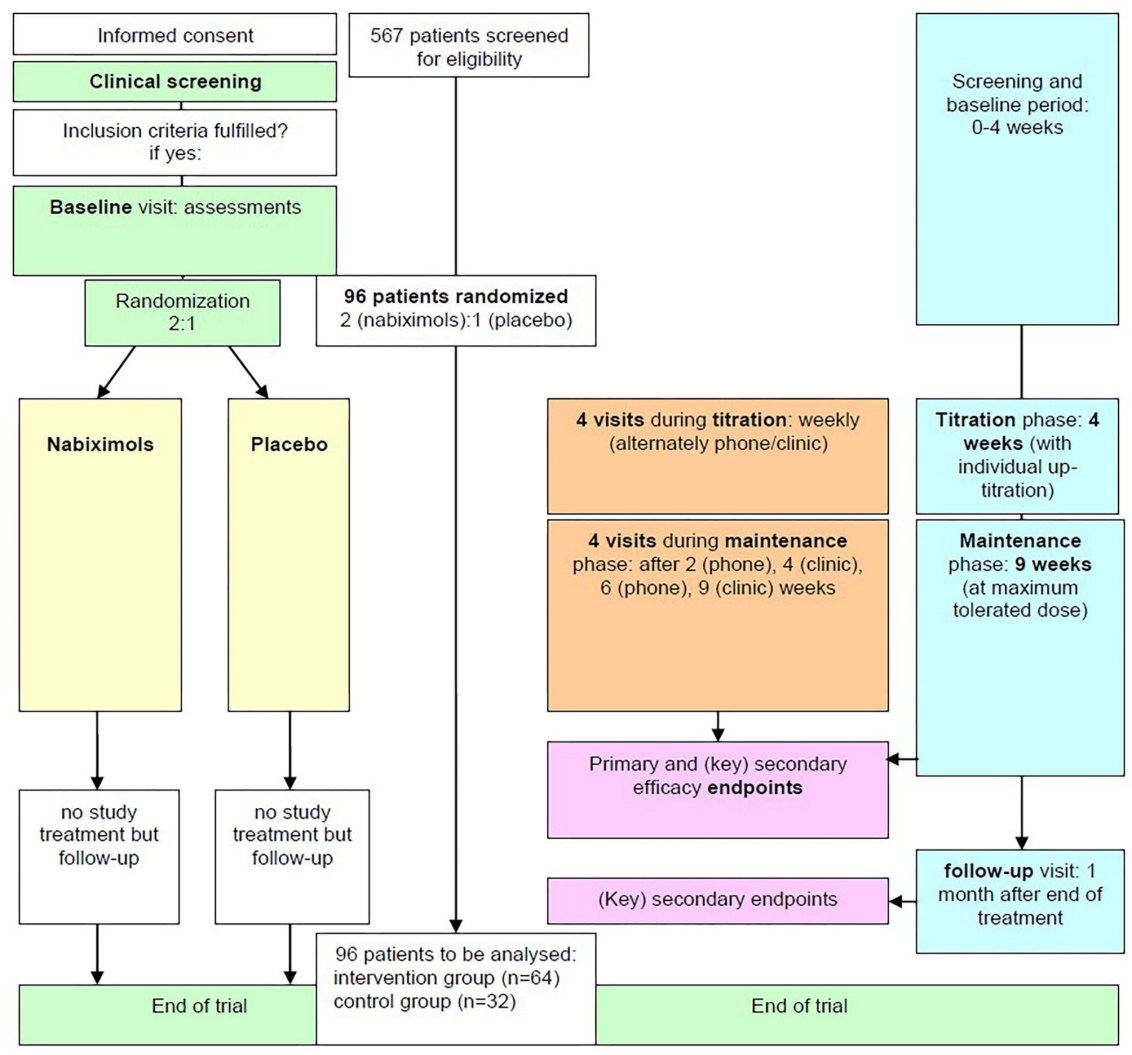
Study Flow. MS, milestone.

This is a multicenter, prospective, randomized, double-blind, placebo controlled, parallel-group, phase IIIb investigator-initiated clinical trial. The time from first patient in to last patient out is expected to be 54 months including a recruitment period of ~28 months. The trial duration per patient will be about 17 weeks, including a 4 weeks up-titration, a 9 weeks maintenance phase, and 4 weeks of follow-up. The study flow is displayed on Figure 1.

**Figure 2:**
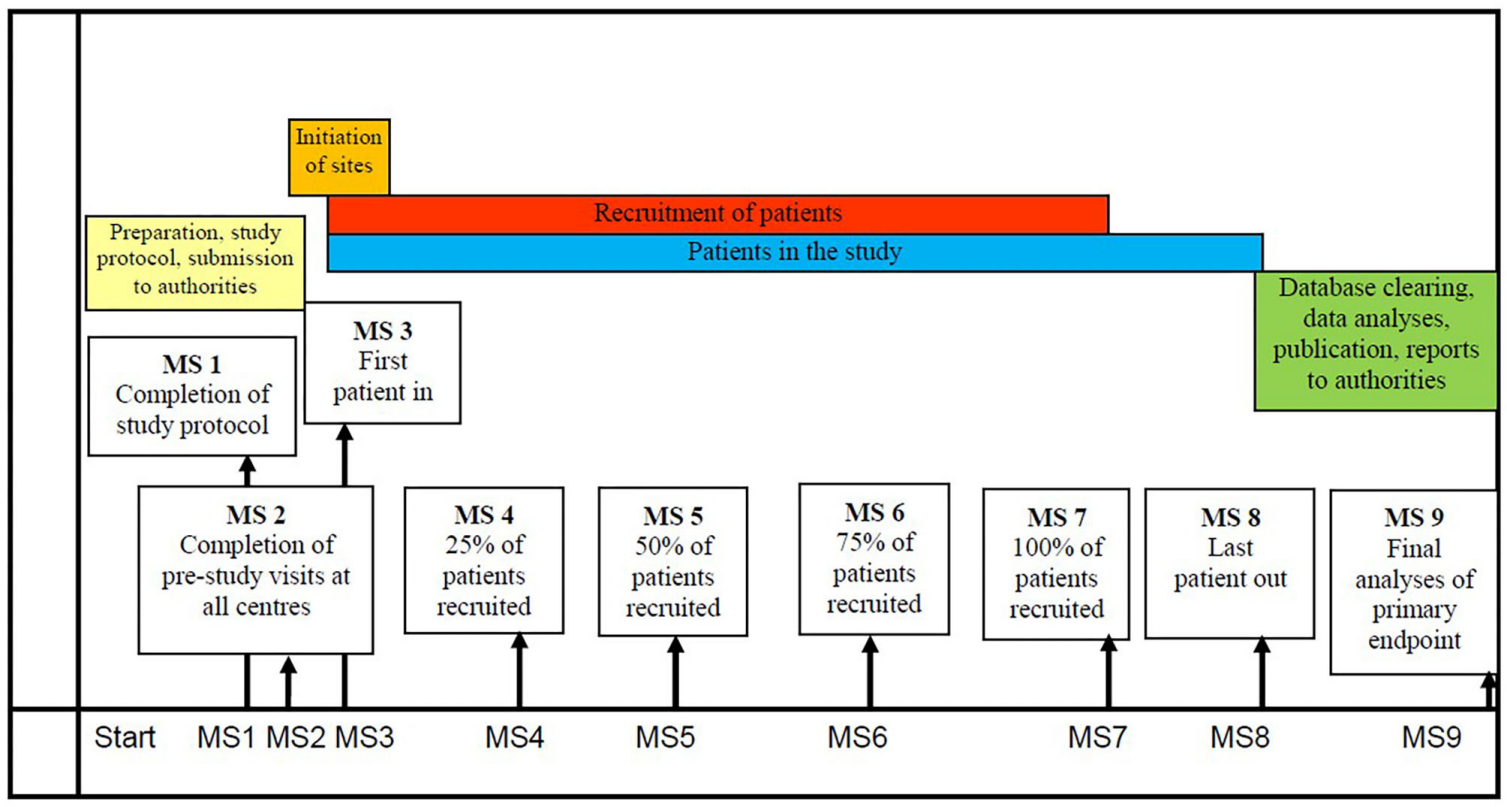
Study Design.

Patients will be randomized to receive nabiximols or placebo with a ratio of 2:1 over the course of a 13-weeks double-blind treatment period. The treatment plan is identical for both treatment arms. In the placebo arm, patients will be treated with a placebo spray identical to nabiximols in visual appearance, taste, and odor. The study design is displayed on Figure 2.

### Recruitment

A total number of 96 eligible patients will be included in the clinical trial across 6 participating German study sites. Study participants will be mainly recruited through the study centers' outpatient clinics. In addition, the study will be announced in German self-help and advocacy groups, newsletters and annual meetings. During a screening visit, full information on the study design and the study medication (orally and in writing) will be provided. Before enrollment, a written informed consent will be obtained. Travel costs related to the study participation will be reimbursed. No further financial compensation will be offered.

### Eligibility Criteria

The following inclusion criteria were defined:

- TS or chronic motor tic disorder (CMT) according to DSM-5 (CTD = TS + CMT)
- Age ≥ 18 years
- Yale Global Tic Severity Scale – Total Tic Score (YGTSS-TTS) > 14 for patients with TS or YGTSS-TTS > 10 for patients with CMT
- Clinical Global Impression Scale – Severity of Illness (CGI-S) ≥ 4
- If the patient will be on any medication (or deep brain stimulation) for tics or comorbidities, a stable dose will have to be obtained at least 30 days before entering the study and maintained during the study
- Signed written informed consent
- Capacity to understand the investigational nature, potential risks and benefits of the clinical trial
- Women of childbearing potential will need to test negative to a beta human chorionic gonadotropin (β-HCG) pregnancy test at screening
- Both sexually active men and women of child-bearing potential will need to agree to use valid methods of contraception.

The following exclusion criteria were defined:

- Psychiatric comorbidities in primary need of treatment
- Ongoing behavioral treatment for tics
- History of schizophrenia, psychosis, severe personality, or pervasive developmental disorder
- Presence of suicidal ideation (intent or plan) within the last 12 months
- Current diagnosis of substance abuse or dependence
- Secondary tic disorders and other significant neurological disorders
- Current or past severe cardiovascular diseases, hepatitis C, or other severe hepatic and renal disorders
- Any medical condition that might interfere with the patient's participation in the study or pose a risk for the patient
- Use of cannabis or any cannabis-based medicine in the 30-day period prior to study entry and/or positive THC urine test at baseline
- Positive pregnancy test
- Pregnancy or lactation period
- Participation in any investigational medication study within 30 days prior to study entry or planned medication change during the study period
- Known or suspected hypersensitivity to any of the active substances or any excipients of the investigational medicinal product.

### Blinding and Randomization

This is a double-blind clinical trial. To prevent unblinding, THC blood tests during the treatment phase will be sent to the laboratory of an unaffiliated institution (University Medical Center Mainz) and the results will be kept confidentially until the end of the study. In addition to examiner and self-assessments for tics, we will use a video-based tic rating as a secondary outcome measure. Since video evaluation will be done centrally by otherwise uninvolved blinded raters, this assessment will be robust against unintentional unblinding.

A permuted block randomization will be used to randomize the treatment allocation in a 2:1 ratio (nabiximols:placebo). Randomization will be conducted centrally and will be stratified by center. We decided in favor of a 2:1 randomization because uneven allocation (i) allows for more safety information in the active treatment group, (ii) enables more precise response rate estimation in the active treatment group, and (iii) facilitates patient recruitment. This study is sufficiently powered for a 2:1 randomization with a power of 90%.

### Compliance

Medication (nabiximols and placebo) will be dispensed to the patients in limited amounts at each clinic visit. Empty spray bottles will be collected at clinic visits. At each clinical visit, a THC urine test will be done to check for compliance with the study medication on the one hand and any concomitant use of cannabis on the other hand (to avoid unblinding, analyses will be done centrally at the end of the trial).

### Active Treatment Phase

The titration phase will last 4 weeks in all patients, independently of the maximum dose and the time for up-titration. The starting dose will be 1 spray/day (= 100 μl spray = 2.7 mg THC/2.5 mg CBD). The standard dose escalation will be the following: For the first 4 days, dose can be increased by 1 spray every 2 days, and thereafter by 1 spray every day up to a maximum dose of 12 sprays/day (= 1,200 μl spray = 32.4 mg THC/30 mg CBD). However, depending on individual tolerability and efficacy slower dose increase is possible. Patients will be allowed to increase their dose to achieve sufficient efficacy in both, the active and the placebo - treatment group to reflect that due to high inter-individual variability different patients may require different doses. Since no target dose is defined, dosage can be increased until the patient reaches his/her individually tolerated maximum dose (1–12 sprays/day) based on the patient's judgment and investigator's assessments. Different dose levels thus reflect different needs of patients with different patient characteristics and not a systematic dose-response investigation. For patients in the placebo arm, titration will follow the same scheme as for nabiximols.

After the 4-weeks titration phase, treatment will be continued at a stable dosage for another 9 weeks. However, dose adjustment will be possible. Thereafter, medication will be withdrawn without down-titration.

### Study Visits and Assessment Instruments

A full assessment schedule for all study and phone visits is given in detail on Table 1.

**Table 1:** Schedule of Study Assessments and Visits.

	**Screening**	**Base-line**	**Titration phase**				**Maintenance phase**				**Follow-up**
**Visit**	1	2	3	4	5	6	7	8	9	10	11
Clinic visits	X	X		X		X		X		X	X
Phone visits			X		X		X		X		
**Week**	−4 to 0	0	1	2	3	4	6	8	10	13	17
**General procedures**											
Written informed consent	X										
Inclusion/exclusion criteria	X										
Demographics	X	X									
Medical & medication history	X	X									
**Laboratory examinations**											
Urine THC	X	(X)^d^									
Blood exocannabinoids and endo-cannabinoids (including THC)		X		X		X		X		X	X
Urine ß-HCG	X	(X)^a^		X		X		X		X	X
**Intervention**											
Randomization	X										
Compliance: asking for compliance			X	X	X	X	X	X	X	X	
**Psychometric assessments**											
Tics: - YGTSS	X	X		X		X		X		X	X
- ATQ		X		X		X		X		X	X
- MRVS		X		X		X		X		X	X
Severity of disease: CGI-S	X	X		X		X		X		X	X
Improvement of disease: CGI-I				X		X		X		X	X
Premonitory urges: PUTS		X		X		X		X		X	X
Quality of life: GTS-QoL		X		X		X		X		X	X
Mood: BDI-II		X		X		X		X		X	X
Anxiety: BAI		X		X		X		X		X	X
ADHD: - DSM-IV symptom list		X									
- WURS-k		X									
- CAARS		X		X		X		X		X	X
OCD: Y-BOCS		X		X		X		X		X	X
Sleep quality: PSQI		X		X		X		X		X	X
Impulsivity: I-8		X		X		X		X		X	X
Patient health: SF-12		X		X		X		X		X	X
Rage attacks: RAQ		X		X		X		X		X	X
**Fitness to drive:**											
- Patient's specific traffic medical history^c^		X^c^									
- Self-assessment for driving ability		X^c^								X^c^	
- Test battery Fa. Schuhfried		X^c^								X^c^	
Evaluation of neurological impairment and impact on driving ability		X		X		X		X		X	X
**Adverse events**											
Open questions			X	X	X	X	X	X	X	X	X
Blood pressure, heart rate		X		X		X		X		X	X
C-SSRS assessment^b^		X		X		X		X		X	X

*THC, delta-9-tetrahydrocannabinol; ß-HCG, beta human chorionic gonadotropin; YGTSS, Yale Global Tic Severity Scale; ATQ, Adult Tic Questionnaire; MRVS, Modified Rush Video-Based Tic Rating Scale; CGI-S, Clinical Global Impression Scale – Severity of Illness; CGI-I, Clinical Global Impression – Improvement Scale; PUTS, Premonitory Urge of Tics Scale; GTS-QoL, Gilles de la Tourette Syndrome-Quality of Life Scale; BDI, Beck Depression Inventory; BAI, Beck Anxiety Inventory; ADHD, Attention-Deficit-Hyperactivity Disorder; DSM, Diagnostic and Statistical Manual of Mental Disorders; WURS-k, Wender Utah Rating Scale; CAARS, Conners' Adult ADHD Rating Scale; OCD, Obsessive-Compulsive Disorder; Y-BOCS, Yale-Brown Obsessive Compulsive Scale; PSQI, Pittsburgh Sleep Quality Index; I-8, Skala Impulsives-Verhalten-8 (measure of impulsivity); SF-12, 12-item short-form Health Survey; RAQ, Rage Attacks Questionnaire*.

a *Only if screening and baseline visit are at least 2 weeks apart*.

b *C-SSRS (Columbia-Suicide Severity Rating Scale): The Baseline version is used at the Screening visit and the Since Last Visit version at all subsequent visits*.

c *In patients not recruited at MHH or LMU, who participate in the study part “Fitness to drive,” variations of the scheduled baseline and follow-up “Fitness to drive” visits are possible*.

d *Only if screening and baseline visit are at least 2 weeks apart*.

#### Assessment Instruments

A test battery will be administered at clinical visits:

- Tics: YGTSS (16), Modified Rush Video-Based Tic Rating Scale (MRVS) (17), and Adult Tic Questionnaire (ATQ), a tic self-rating scale, which is parallel in format and content to the Parent Tic Questionnaire (18)
- Premonitory urges related to tics: Premonitory Urge of Tics Scale (PUTS) (19)
- Psychiatric comorbidities: Beck Depression Inventory (BDI-II) (20), Beck Anxiety Inventory (BAI) (21), Conners' Adult ADHD Rating Scale (CAARS) (22), DSM-IV symptom list, Wender Utah Rating Scale (WURS-k) (23), Yale-Brown Obsessive Compulsive Scale (Y-BOCS) (24, 25), Rage Attacks Questionnaire (RAQ) (26), Skala Impulsives-Verhalten-8 (I-8) (27), and Pittsburgh Sleep Quality Index (PSQI) (28)
- Quality of life and overall impairment and severity of disease: Clinical Global Impression-Severity (CGI-S) (29), Gilles de la Tourette Syndrome-Quality of Life Scale (GTS-QoL) (30) and the 12-item short-form Health Survey (SF-12) (31)
- Safety assessments including the Columbia-Suicide Severity Rating Scale (C-SSRS) Baseline version (32)
- Driving test “Fitness to drive” (for details see 2.8.2).

In addition, blood will be drawn to measure levels of endocannabinoids and exocannabinoids.

#### Driving Test “Fitness to Drive”

The study part “Fitness to drive” has two main objectives: assessing the driving ability in subjects with TS/CMT in general and investigating the impact of nabiximols on driving ability in this group of subjects. At baseline, therefore, the following additional assessments will be performed: (i) subject's specific traffic medical history, (ii) self-assessment of subjects' general and current driving ability, and (iii) objective assessment of psychomotor skills using The Vienna Test System, a validated, CE-marked and well-established assessment in Germany, which will be carried out in accordance with the German driving license regulations.

“Fitness to drive” is conceived as a dichotomous criterion and is based on the guidelines of the German Federal Highway Research Institute (BASt). A subject will be considered unfit to drive, if he or she has a percent rank below 16 of at least one of the following tests: Reaction time and choice reaction (RT), Stress Behavior capacity (DT-Auslassungen), Stress Behavior performance quantity (DT-Mengenleistung), Concentration (COG), and Perceptual speed (ATAVT).

Data acquisition is carried out during the baseline visit and at visit 10 (after 9 weeks stable treatment) or before discontinuation of the study medication. In addition, at both baseline and follow-up “Fitness to drive” visits, subjects will be asked about their self-evaluation assessment of their general and current driving ability.

For organizational reasons, this study part will be carried out in only two study centers: Hanover (MHH) and Munich (LMU). For patients recruited at other study centers, a participation will be possible (if desired), but will entail additional study visits at MHH or LMU.

### Outcome Measures

#### Primary Endpoints

The main objective of this study is to demonstrate that treatment with nabiximols is superior to placebo in patients with TS/CMT. The primary outcome variable will be response to treatment according to YGTSS-TTS, defined as a reduction in YGTSS-TTS of at least 30% (compared to baseline) after 9 weeks of stable treatment.

#### Secondary Endpoints

Key secondary analyses will be performed on the continuous YGTSS-TTS changes from baseline with an ANCOVA model adjusted for the baseline YGTSS-TTS and center. Further secondary outcomes are improvements on other clinical variables: YGTSS-Global Score (sum of TTS and global impairment), MRVS, CGI-I, CGI-S, ATQ, GTS-QoL, PUTS, BDI-II, Y-BOCS, CAARS, BAI, PSQI, I-8, SF-12, and RAQ. These measurements are recommended for clinical trials in TS in order to assess the full spectrum of the disease including psychiatric comorbidities and patients' quality of life. Besides the YGTSS-TTS we will use the MRVS - an observational examiner tic assessment - as well as the ATQ, which is a self-assessment scale for tics. In addition, the YGTSS Global score takes into account the overall tic-related impairment on a patient's life, similar to the CGI-I and CGI-S that measure overall clinical impairment and improvement. Finally, the GTS-QoL is used to assess disease specific quality of life. The PUTS is an instrument that measures premonitory urges often preceding the tics. To assess comorbid pathologies, we will use the BDI-II to assess depression, Y-BOCS for obsessions and compulsions, CAARS for ADHD, BAI for anxiety, PSQI for sleep disturbances, I-8 for impulsivity, RAQ for rage attacks, and SF-12 for overall health. Other secondary endpoints are the outcome variables from the study part on driving ability “Fitness to drive.”

#### Safety Assessments, Quality Assurance and Ethics

Safety assessments include (serious) (S)AEs, C-SSRS, blood pressure, and pulse at each clinic visit. With respect to subject's driving ability, neurological and psychological impairment will be evaluated by a psychologist and a research physician and documented at each clinic visit.

To assure data quality and patients' safety, regular monitoring visits will be performed and an independent data monitoring committee (DMC) will be established. The trial will be conducted following the principles of ICH-GCP, the German Drug Law (AMG), and the Declaration of Helsinki. Study protocol including possible amendments, patient information with consent and substantial amendments will be/were approved by the responsible ethics committees and the federal authorities.

### Sample Size Calculation

The sample size calculation is based on a previous trial, in which 24 patients with TS had been randomized to 6 weeks of THC or placebo medication with a ratio of 1:1 (drop-out rate 17%: THC *n* = 3, placebo *n* = 1) (8). Under the assumption that the relative reduction on YGTSS-TTS is normally distributed in each treatment group, the probabilities for a reduction of at least 30% (criteria for treatment response) were calculated as 0.010 for placebo and 0.294 for the active arm. These calculations refer to an intention-to-treat (ITT) population, where missing values for YGTSS-TTS response at the end of treatment were set to non-response. This approach was chosen due to its higher robustness in regard to small datasets, obtaining more conservative values than the observed responder rates. The sample size calculations were conducted using nQuery 7.0 software and are based on an exact Fisher-test with a two-sided significance level of 5%, a power of 90%, and the above-mentioned responder rates (placebo = 0.010, THC = 0.294). Under a randomization ratio of 2:1, the resulting group sizes were *n* = 50 for nabiximols and *n* = 25 for placebo. To compensate for a potentially diminished treatment effect due to self-medication, non-compliance, and drop-out, we decided to increase the calculated sample size by ~30%. Thus, we decided to include 64 patients in the active arm and 32 in the placebo arm, adding up to a total sample of 96 patients.

### Statistical Analysis

#### Primary Analysis

The primary analysis will be carried out in the ITT population and use the YGTSS-TTS as criterion for a binary responder variable. A patient will be considered a responder, if a >30% decrease in YGTSS-TTS is observed as compared to baseline. The primary analysis of the responder proportion will be done with a Mantel-Haenszel estimate for the risk difference (active treatment minus placebo) stratified by center. If the lower bound of the corresponding 95%-confidence interval is above 0, superiority of the active treatment over placebo as assessed by YGTSS-TTS responder criterion is demonstrated. The two-sided type-I-error rate will be set to 5%.

#### Key Secondary Analysis

If the primary null hypothesis will be rejected and superiority of the active treatment over placebo will be demonstrated, non-inferiority of the active treatment compared to placebo will be hierarchically tested regarding the proportion of patients' fitness to drive at the 2-sided significance level of 5% in all patients. The analyses will be done with a Mantel-Haenszel estimate for the risk difference (active treatment minus placebo) stratified by center. If the lower bound of the corresponding 95%-confidence interval is above the non-inferiority margin of −32%, non-inferiority of the active treatment over placebo regarding fitness to drive will be concluded.

#### Secondary and Safety Analyses

As secondary analysis, we will use YGTSS-TTS change from baseline at 9 weeks of treatment as continuous outcome variable. An ANCOVA will be computed with the covariates: baseline YGTSS-TTS and center. Additionally, a mixed model will be carried out to assess longitudinal changes in YGTSS-TTS (measured by YGTSS-TTS change from baseline to week 4 and week 9). The model will include repeated measures with a first-order autoregressive covariance structure, and include baseline YGTSS-TTS and center as covariates. For this model, the missing values will not be replaced. Further secondary endpoints will be analyzed with the same statistical methods. All secondary analyses are exploratory. Further exploratory analyses may investigate, whether the finally required dose can be related to baseline patient characteristics so that dose recommendations could be given to the patients.

Absolute and relative frequencies of (S)AEs will be calculated for the full analysis set and will be compared between treatment groups by using a chi-squared test.

## Discussion

### Clinical Implications

This will be the first well-powered controlled clinical trial investigating efficacy and safety of nabiximols in patients with TS/CMT. Thus, this study is not only the very first large controlled study in patients with tic disorders using a cannabis-based medicine, but also the first large controlled trial in a (hyperkinetic) movement disorder in general. Since we will also assess the effects of nabiximols on a variety of psychiatric symptoms (including ADHD, OCD, depression, and anxiety), this study will provide urgently needed data on the potential use of cannabis-based medicines in these conditions (33). This study will be of enormous health-economic relevance, because a substantial number of patients with TS/CMT (but also other psychiatric diseases such as ADHD) uses cannabis as self-medication. However, the current data basis is weak and, therefore, most physicians do not recommend cannabis-based medicines for their patients. Finally, this study will address an important practical question, whether treatment with nabiximols impairs driving ability in patients with TS/CMT.

We hypothesize that nabiximols will be effective not only in the treatment of tics, but also in a large spectrum of psychiatric comorbidities improving patients' quality of life. Accordingly, we expect that patients' driving ability will not be worsened by treatment with nabiximols. If these assumptions are to be proven correct, nabiximols would be a valuable treatment alternative in adult patients with otherwise treatment resistant TS/CMT.

### Underlying Mechanisms

Several lines of evidence suggest a dopaminergic hypothesis in TS. More precisely, a dysbalance in presynaptic tonic and phasic dopamine is assumed to underly the pathophysiology of tics. However, there is also evidence for an involvement of other neurotransmitter systems and alterations in the dopaminergic system cannot explain the broad spectrum of psychiatric comorbidities seen in most patients with TS. Alternatively to the dopaminergic hypothesis, it can be speculated that TS is caused by a dysfunction in the endocannabinoid system (ECS), since the ECS is the most important neuromodulatory system in the brain. In line with this assumption, changes in cerebrospinal fluid (CSF) endocannabinoid levels have been reported (34). However, based on the complex interaction between the endocannabinoid and the dopaminergic system, it can also be speculated that stimulation of the ECS by use of exocannabinoids may attenuate dopaminergic hyperinnervation.

### Limitations

Our study has the following possible limitations: (i) nabiximols constitutes a specific formulation of cannabis extract with a 1:1 ratio of THC:CBD and, therefore, results may not be generalized to all cannabis-based medicines; (ii) we defined 12 sprays of nabiximols as maximum dose, since this is the maximum dose licensed for the treatment of spasticity in multiple sclerosis. Theoretically, this maximum dose might be too low for patients with TS/CMT; (iii) although we decided for a quite long treatment period of 13 weeks (plus a follow-up visit), from this study no conclusions can be drawn on long-term effects; (iv) due to methodological limitations, the unlikely case of additional recreational use of cannabis in the nabiximols group cannot entirely be excluded; (v) theoretically, patients can unblind themselves by a THC test; (vi) recruitment and results might be biased by patients with prior use of cannabis; (vii) we used a 2:1 randomization scheme that has a lower power compared to a 1:1 randomization. Nevertheless, we plan on compensating for this with a higher sample size; (viii) driving performance can be more realistically assessed in on-the-road-tests than in an experimental design as used in this study; (ix) participation in the study part “fitness to drive” is only mandatory for a subgroup of patients in only two of six study sites.

## Ethics Statement

The trial will be conducted following the principles of ICH-GCP, the German Drug Law (AMG), and the Declaration of Helsinki. The study protocol including amendments, patient information with consent and substantial amendments was approved by the ethics committee of Hannover Medical School (MHH) as the main committee and in addition by the ethic committees of all participating centers: the University of Lübeck, the Ludwig Maximilian University of Munich (LMU), the RWTH Aachen University, the University of Cologne and the University of Freiburg as well as the federal authorities.

## Author Contributions

The first draft of this manuscript was written by EJ. AP contributed background information needed for the first draft of the manuscript, in addition AP is in charge of the study part Fitness to drive. AP, CF, MH, MM, CSchu and EJ are responsible for study assessments at MHH. CC was responsible for study assessments at LMU. AB and CC offered their expertise in the assessment of Fitness to drive. BL and LB helped with the study design and will measure levels of exo- and endocannabinoids. AK, LA, and SH contributed in the power calculation, determining the randomization protocol, and the selection and planning of the statistical analysis. MM helped at shaping the research protocol and at getting the study up and running. CShi was deputy PI and provided supervision and guidance to the team and input in coordinating and conducting the trial. KM-V was the PI of this study and contributed on all stages of the development of the trial and is the senior author of this article. All authors provided critical feedback and contributed to the final manuscript.

## Conflict of Interest

KM-V has received financial or material research support from the EU (FP7-HEALTH-2011 No. 278367, FP7-PEOPLE-2012-ITN No. 316978), the German Research Foundation (DFG: GZ MU 1527/3-1), the German Ministry of Education and Research (BMBF: 01KG1421), the National Institute of Mental Health (NIMH), the Tourette Gesellschaft Deutschland e.V., the Else-Kroner Fresenius-Stiftung, and GW, Abide Therapeutics, Lundbeck, Syneos Health, Therapix Biosciences Ltd, Almirall Hermal GmbH, GW pharmaceuticals. She has received consultant's honoraria from Abide Therapeutics, Tilray, Resalo Vertrieb GmbH, Columbia Care, Bionorica Ethics GmbH, Lundbeck and Eurox Deutschland GmbH. She is a consultant or advisory board member for Abide Therapeutics, Alirio, The Academy of Medical Cannabis Limited, CannaMedical Pharma GmbH, CannaXan GmbH, Columbia Care, Canopy Growth, Leafly Deutschland GmbH, Lundbeck, Nomovo Pharm, Nuvelution TS Pharma Inc., Resalo Vertrieb GmbH, Sanity Group, Syqe Medical Ltd., Therapix Biosciences Ltd., Tilray, Wayland Group, Zynerba Pharmaceuticals, and CTC Communications Corporation. She has received speaker's fees from Tilray, Wayland Group, Emalex, Eurox group, PR Berater, Aphria, Ever pharma GmbH, and Cogitando GmbH. She has received royalties from Medizinisch Wissenschaftliche Verlagsgesellschaft Berlin, Elsevier, and Kohlhammer. She holds shares of Nomovo Pharm. She served as a Guest editor for Frontiers in Neurology on the research topic “The neurobiology and genetics of Gilles de la Tourette syndrome: new avenues through large-scale collaborative projects,” is Associate editor for “Cannabis and Cannabinoid Research” and Editorial Board Member for “Medical Cannabis and Cannabinoids.” The remaining authors declare that the research was conducted in the absence of any commercial or financial relationships that could be construed as a potential conflict of interest.
